# Co-infection of Long-Term Carriers of *Plasmodium falciparum* with *Schistosoma haematobium* Enhances Protection from Febrile Malaria: A Prospective Cohort Study in Mali

**DOI:** 10.1371/journal.pntd.0003154

**Published:** 2014-09-11

**Authors:** Safiatou Doumbo, Tuan M. Tran, Jules Sangala, Shanping Li, Didier Doumtabe, Younoussou Kone, Abdrahamane Traoré, Aboudramane Bathily, Nafomon Sogoba, Michel E. Coulibaly, Chiung-Yu Huang, Aissata Ongoiba, Kassoum Kayentao, Mouctar Diallo, Zongo Dramane, Thomas B. Nutman, Peter D. Crompton, Ogobara Doumbo, Boubacar Traore

**Affiliations:** 1 Mali International Center of Excellence in Research, University of Sciences, Techniques, and Technology of Bamako, Bamako, Mali; 2 Laboratory of Immunogenetics, National Institute of Allergy and Infectious Diseases, National Institutes of Health, Rockville, Maryland, United States of America; 3 Division of Biostatistics and Bioinformatics, Sidney Kimmel Comprehensive Cancer Center, Johns Hopkins University, Baltimore, Maryland, United States of America; 4 Laboratory of Parasitic Diseases, National Institute of Allergy and Infectious Diseases, National Institutes of Health, Bethesda, Maryland, United States of America; Swiss Tropical and Public Health Institute, Switzerland

## Abstract

**Background:**

Malaria and schistosomiasis often overlap in tropical and subtropical countries and impose tremendous disease burdens; however, the extent to which schistosomiasis modifies the risk of febrile malaria remains unclear.

**Methods:**

We evaluated the effect of baseline *S. haematobium* mono-infection, baseline *P. falciparum* mono-infection, and co-infection with both parasites on the risk of febrile malaria in a prospective cohort study of 616 children and adults living in Kalifabougou, Mali. Individuals with *S. haematobium* were treated with praziquantel within 6 weeks of enrollment. Malaria episodes were detected by weekly physical examination and self-referral for 7 months. The primary outcome was time to first or only malaria episode defined as fever (≥37.5°C) and parasitemia (≥2500 asexual parasites/µl). Secondary definitions of malaria using different parasite densities were also explored.

**Results:**

After adjusting for age, anemia status, sickle cell trait, distance from home to river, residence within a cluster of high *S. haematobium* transmission, and housing type, baseline *P. falciparum* mono-infection (n = 254) and co-infection (n = 39) were significantly associated with protection from febrile malaria by Cox regression (hazard ratios 0.71 and 0.44; *P* = 0.01 and 0.02; reference group: uninfected at baseline). Baseline *S. haematobium* mono-infection (n = 23) did not associate with malaria protection in the adjusted analysis, but this may be due to lack of statistical power. Anemia significantly interacted with co-infection (*P* = 0.009), and the malaria-protective effect of co-infection was strongest in non-anemic individuals. Co-infection was an independent negative predictor of lower parasite density at the first febrile malaria episode.

**Conclusions:**

Co-infection with *S. haematobium* and *P. falciparum* is significantly associated with reduced risk of febrile malaria in long-term asymptomatic carriers of *P. falciparum*. Future studies are needed to determine whether co-infection induces immunomodulatory mechanisms that protect against febrile malaria or whether genetic, behavioral, or environmental factors not accounted for here explain these findings.

## Author Summary

The parasitic diseases malaria and schistosomiasis are tremendous public health burdens, each affecting over 200 million people worldwide with substantial geographic overlap in sub-Saharan Africa. Understanding how schistosomiasis influences the human immune response to *Plasmodium*, the agent of malaria, can be important for developing effective malaria vaccines. Past studies have tried to determine if infection with *Schistosoma haematobium*, which causes urinary schistosomiasis, affects the number of febrile attacks from malaria caused by *Plasmodium falciparum* in communities where the diseases overlap, but the findings have been inconsistent. Here, we examined 616 healthy people from a village in Mali for symptomless infections with *S. haematobium* and treated those with infections. We then followed them over a single malaria-transmission season of 7 months during which we diagnosed and treated all febrile malaria attacks. After the season, we examined archived blood collected at enrollment to look for occult *P. falciparum* infection. The study revealed that people who were infected with both parasites at the beginning of the season were better protected from the malaria attacks than those who were uninfected or infected with either parasite alone. Further studies are needed to confirm these findings and to determine the biological basis for this phenomenon.

## Introduction

Malaria and schistosomiasis, caused by the protozoan *Plasmodium* and the trematode helminth *Schistosoma*, respectively, impose tremendous public health burdens in tropical and subtropical countries. Whereas malaria afflicts ∼210 million people annually, with ∼0.6 million malaria deaths in 2012 caused primarily by *Plasmodium falciparum* in sub-Saharan Africa [1], *Schistosoma* infects ∼240 million people annually, with >90% of cases occurring in Africa [2]. In humans, schistosomiasis manifests as chronic inflammation around schistosome eggs that are embedded within host tissues. Specifically, urogenital schistosomiasis, caused by *Schistosoma haematobium*, affects the ureteral or bladder wall and can lead to hematuria-induced anemia, urogenital deformities, bladder cancer, and diminished health-related quality of life [3]. The substantial epidemiological overlap of these two parasitic infections invariably results in frequent co-infections [4]. The challenges facing the development of a highly effective malaria vaccine have generated interest in understanding the interactions between malaria and co-endemic helminth infections, such as those caused by *Schistosoma*, that could impair vaccine efficacy by modulating host immune responses to *Plasmodium* infection [5].

Both malaria and schistosomiasis are endemic to Mali, a landlocked country in West Africa with a population of 14.9 million. Intense, seasonal transmission of malaria occurs over much of the country, with ∼2.1 million malaria cases reported in 2012 [1]. Malaria control strategies include distribution of insecticide-treated bed nets, indoor residual spraying, intermittent preventative therapy, and active case detection of febrile cases at the community level [1]. From 2004–2006, the overall *S. haematobium* prevalence in Mali was 38.3% but varied widely by region [6], and attempts to control the disease with mass drug administration (MDA) with praziquantel have been ongoing since 2005—initially through the Schistosomiasis Control Initiative and then as part of an integrated, national Neglected Tropical Disease (NTD) control program [7].

In co-endemic settings such as Mali, the impact of *S. haematobium* and *P. falciparum* co-infection on the risk of clinical malaria remains unclear. Independent studies have shown that *S. haematobium* co-infection can either correlate positively [8], [9] or negatively [10]–[12] with *P. falciparum* parasite density. Although baseline *S. haematobium* infection decreased the risk of febrile malaria in a prospective cohort study of Malian children [10], it did not alter malaria risk in a malaria vaccine efficacy trial of Kenyan children in which all children received curative treatment immediately prior to the surveillance period [13]. One possible explanation for this discrepancy is confounding by asymptomatic *P. falciparum* carriage at enrollment, which has been associated with a decrease in the subsequent risk of febrile malaria [14], [15] and likely accounted for a significant proportion of children in the Malian study [10] but not the Kenyan study [13]. Additional factors that have been shown to associate with both urogenital schistosomiasis and malaria while possibly affecting subsequent malaria outcomes are co-infection with helminths other than *S. haematobium* [13], [16], iron-deficiency anemia [17]–[20], and contextual factors related to geography and ecology [9], [21], [22].

To clarify the relationship between urinary schistosomiasis and malaria, we evaluated the effect of baseline *S. haematobium* mono-infection, asymptomatic *P. falciparum* carriage (baseline *P. falciparum* mono-infection) at the end of the six-month dry season, and co-infection with both parasites on the risk of febrile malaria in a prospective cohort study of Malian children and adults living in an area where both diseases are co-endemic. Individuals diagnosed with urogenital schistosomiasis were treated with praziquantel within 6 weeks of enrollment, prior to the peak of the malaria transmission season. We adjusted for possible confounders of malaria risk, including age, sickle cell trait (HbAS), anemia, and spatial factors as determined by distance from home to river and residence within a cluster of high *S. haematobium* transmission.

## Methods

### Ethics Statement

The Ethics Committee of the Faculty of Medicine, Pharmacy and Dentistry at the University of Sciences, Techniques, and Technology of Bamako, and the Institutional Review Board of the National Institute of Allergy and Infectious Diseases, National Institutes of Health approved this study (ClinicalTrials.gov identifier: NCT01322581). Written, informed consent was obtained from adult participants and from the parents or guardians of participating children.

### Study Site

The study was conducted in the village of Kalifabougou, Mali, which is located 40 km northwest of Bamako, Mali. Kalifabougou is in the savanna ecoclimatic zone where annual rainfall is 800–1,200 mm per year. Among its inhabitants, Bambara is the predominant ethnic group, and ∼90% of residents engage in subsistence farming. Malaria transmission is intense and seasonal, occurring from June through December, with the vast majority of malaria cases caused by *P. falciparum* [23]. *Schistosoma haematobium* is also endemic in this region of Mali, with peak transmission occurring during the dry season from January through March when temporary water sources serve as ideal breeding sites for snails, which are the intermediate hosts for schistosomes. Schistosomiasis control in Kalifabougou is done primarily via case treatment and MDA with praziquantel as part of a national integrated NTD control program [7]. Overall *S. haematobium* prevalences in nearby communes were 12.9% in Kati in 2005 (data from the Malian national NTD control program) and 6% in Kambila in 2006 [15].

### Study Population and Procedures

**Figure 1:**
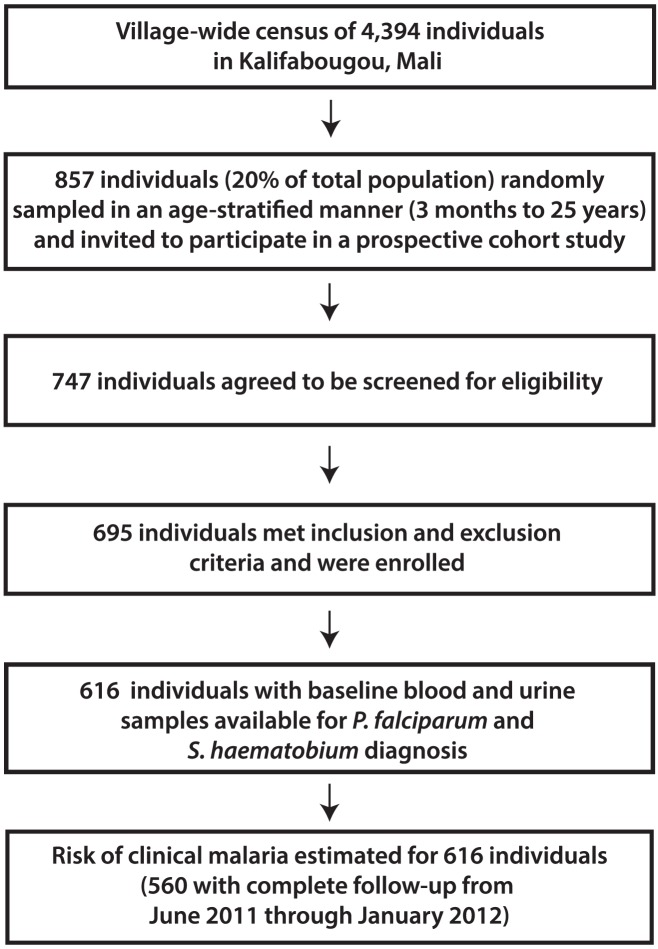
Study participants and risk analysis flow chart.

The study population has been previously described [23], [24]. Enrollment procedures are summarized in Figure 1. In July 2010, prior to the start of this study, we conducted a village-wide census of the Kalifabougou study site and determined the total population to be 4,394. Using the complete census data, we then randomly sampled census ID numbers in an age-stratified manner (age 3 months to 25 years) and invited these individuals or their parents/guardians to be screened for participation in the study. Of the 857 individuals who were invited, 747 (87%) agreed to be screened for eligibility. Of the 747 individuals who were screened for eligibility, 695 (93%) met the inclusion and exclusion criteria and were enrolled in May 2011. Exclusion criteria at enrollment included a hemoglobin level <7 g/dL, axillary temperature ≥37.5°C, acute systemic illness, underlying chronic disease, use of antimalarial or immunosuppressive medications in the past 30 days, or pregnancy. Notably, only 29 individuals (4% of all individuals screened) were excluded on the basis of fever. Baseline hemoglobin values, measured by a HemoCue analyzer, were used to determine anemia status based on WHO criteria [25]. As part of MDA [7], [26], all residents >5 years of age received albendazole, ivermectin, and praziquantel in March 2011 (prior to enrollment) and only albendazole and ivermectin in October 2011.

### Diagnosis and Treatment of Infections

#### Clinical malaria episodes

After enrollment individuals were followed during the ensuing malaria season for 7 months. Clinical malaria episodes were detected prospectively by self-referral and weekly active clinical surveillance visits which alternated between the study clinic and the participants' homes. All individuals with signs and symptoms of malaria and any level of *Plasmodium* parasitemia detected by light microscopy were treated according to the National Malaria Control Program guidelines in Mali. The research definition of clinical malaria was parasitemia of ≥2500 parasites/µL, an axillary temperature of ≥37.5°C within 24 hours, and no other cause of fever discernible by physical exam. The primary endpoint was the time to the first or only febrile malaria episode. We also explored secondary definitions of malaria using parasite density thresholds of ≥500, ≥2500, and ≥5000 parasites/µL.

#### Blood smears

Thick blood smears were stained with Giemsa and counted against 300 leukocytes. Parasite densities were recorded as the number of asexual parasites/µl of blood based on a mean leukocyte count of 7500 cells/µl. Each smear was read in blinded manner by two certified microscopists of the laboratory team.

#### *Schistosoma* and other helminth infections at enrollment

Urine and stool samples were collected from participants at the time of enrollment, and samples were processed within 24 hours of collection. *Schistosoma haematobium* eggs were quantified by microscopy after urine filtration with Nytrel filters (Vestergaard Frandsen) from a single urine specimen. *Schistosoma mansoni* and other geohelminth eggs were detected by microscopy of duplicate fecal thick smears using the Kato-Katz technique [27]. Aliquots of stool were cryopreserved at −80°C for subsequent DNA extraction and multi-parallel, real-time PCR for intestinal nematodes (*Necator americanus*, *Ancylostoma duodenale*, *Trichuris trichiura*, *Ascaris lumbricodes*, and *Strongyloides stercoralis*) as described previously [28]. Individuals diagnosed with urinary schistosomiasis were treated with praziquantel within 6 weeks of enrollment.

### Determining *Plasmodium* Blood-Stage Infections

During the scheduled clinic visits, blood was collected by finger prick every two weeks to prepare dried blood spots on filter paper. Detection of asymptomatic *Plasmodium* infection by PCR was done retrospectively at the end of the surveillance period. Detailed methods for PCR detection have been described [23]. *Plasmodium* positive samples were identified as *P. falciparum*, *P. malariae*, or both (mixed infections). For each participant, PCR was performed on blood samples in chronological order from enrollment onwards until the first *P. falciparum* infection was detected.

### Geographical Information Systems Mapping of Study Area

Geographic coordinates of the study participants' place of residence and the major communal buildings, main roads, and large streams in Kalifabougou were determined using GeoXM global positioning system (GPS) receivers (Trimble). Mapping and determination of distances were performed using ArcView 8.0 software (Esri) and QGIS version 2.0.1 (http://www.qgis.org/; map provider: glovis.usgs.gov).

### Statistical Methods

Differences in the baseline characteristics between the *S. haematobium* positive and negative groups (Table 1) and attrition rates were assessed by Fisher's exact test. Linear trends in proportions were assessed by the Cochran-Armitage trend test, whereas differences in means were assessed by Welch's t test. The likelihood ratio test [29] was used to identify high-transmission spatial clusters for *S. haematobium*, *P. falciparum*, or both parasites at the time of enrollment (May 2011). The Kaplan-Meier survival curve was used to estimate the probability of remaining free of clinical malaria during the surveillance period, and the log-rank test was used to compare the survival curves of different subgroups. The Cox proportional hazards model was applied to evaluate the differences in the risk of febrile malaria between the four subgroups: uninfected (reference group), *S. haematobium* mono-infection, *P. falciparum* mono-infection, and co-infection with *S. haematobium* and *P. falciparum*. The Cox model includes the following potential confounding variables (age and distance are continuous): age (per year increase), closest distance from home to river (largest stream in Kalifabougou; per 100 m increase), HbAS, mild anemia at baseline, residence within a *S. haematobium* high-transmission cluster and presence of a metal roof on the participant's home. We also explored a model in which *S. haematobium* mono-infections at baseline were stratified as light (<10 eggs per 10 ml urine) or heavy (>10 eggs per 10 ml urine) but saw no significant difference in risk between the two groups. Thus, *S. haematobium* mono-infection was treated as binary covariate for all subsequent regression analyses.

**Table 1:** Characteristics of study population stratified by baseline *Schistosoma haematobium* infection status^1^.

Characteristic	*S. haematobium* uninfected	*S. haematobium* infected	All	*P* 2
Sample size	554	62	616	
Age group, *n* (%)				<0.001
3 months to 2 years	99 (17.9)	3 (4.8)	102 (16.6)	
3 to 6 years	122 (22.0)	1 (1.6)	123 (20.0)	
7 to 8 years	176 (31.8)	18 (29.0)	194 (31.5)	
9 to 10 years	124 (22.4)	28 (45.2)	152 (24.7)	
11 to 17 years	17 (3.1)	4 (6.5)	21 (3.4)	
18 to 25 years	16 (2.9)	8 (12.9)	24 (3.9)	
Distance from home to clinic (tertiles), *n* (%)				0.003
<289 m	192 (34.7)	14 (22.6)	206 (33.4)	
289 to 773 m	191 (34.5)	15 (24.2)	206 (33.4)	
774 to 4494 m	171 (30.9)	33 (53.2)	204 (33.1)	
Distance from home to river (tertiles), *n* (%)				0.005
<177 m	193 (34.8)	13 (21.0)	206 (33.4)	
177 to 294 m	188 (33.9)	17 (27.4)	205 (33.2)	
294 to 3796 m	173 (31.2)	32 (51.6)	205 (33.2)	
Female gender, *n* (%)	267 (48.2)	26 (41.9)	293 (47.6)	0.42
Metal roof, *n* (%)	346 (62.5)	44 (71.0)	390 (63.3)	0.21
Mild anemia at baseline, *n* (%)	163 (29.4)	19 (30.6)	182 (29.5)	0.88
Positive *Plasmodium* PCR at baseline, *n* (%)				
*P. falciparum*	254 (45.8)	39 (62.9)	293 (47.6)	0.02
*P. malariae*	23 (4.2)	9 (14.5)	32 (5.2)	0.002
mixed infection	19 (3.4)	8 (12.9)	27 (4.4)	0.003
Positive stool microscopy for helminthic infections^3^, *n* (%)				
*S. mansoni*	0 (0.0)	1 (1.6)	1 (0.16)	0.10
*H. nana*	25 (4.6)	5 (8.0)	30 (4.9)	0.22
Positive stool PCR for helminthic infections^4^, n/total tested in group				
*Ancylostoma duodenale*	0/138	0/34	0/172	ND
*Ascaris lumbroides*	0/114	0/31	0/145	ND
*Necator americanus*	0/197	0/45	0/242	ND
*Strongyloides stercoralis*	0/111	0/31	0/142	ND
*Trichuris trichiura*	0/111	0/31	0/142	ND
Sickle cell trait (HbAS), *n* (%)	51 (9.2)	5 (8.1)	56 (9.1)	1.0

1 Data are shown for individuals with urine samples available at enrollment in May 2011.

2 *P* values were obtained by applying Fisher's exact test to compare baseline characteristics between different *S. haematobium* subgroups.

3 Stool samples available for 607 individuals.

4 PCR performed only on a subset of stool samples.

ND = not done.

Moreover, an interaction term between anemia and *S. haematobium* infection was included in the model given the differential risk of malaria between *S. haematobium* infected individuals with and without anemia. The effect of *S. haematobium* and/or *P. falciparum* infection on log-transformed parasite density (asexual parasites/µl) during first malaria episodes was assessed by multiple linear regression with the following independent variables: HbAS, residence in a *S. haematobium* high-transmission cluster, and anemia as categorical variables; and log transformations of age and distance from clinic as continuous variables. Missing data were assumed to be missing at random. Statistical significance was defined as a 2-tailed *P* value of <0.05. Spatial analyses were performed in SaTScan version 9.2 (http://www.satscan.org/). All other analyses were performed in R version 3.0.2 (http://www.R-project.org).

## Results

### Study Population and Infection Prevalence at Enrollment

Of 695 individuals enrolled, 616 (89%) provided blood and urine samples for *P. falciparum* and *S. haematobium* diagnosis, respectively (Figure 1). Of these, 62 (11%) were microscopy positive for *S. haematobium*, 293 (48%) were PCR positive for *P. falciparum* at enrollment, and 39 (6.3%) individuals were co-infected with both parasites. Individuals with heavy *S. haematobium* infections (>9 eggs/10 ml of urine, n = 13) were no more likely to be co-infected with *P. falciparum* than those with light infections (1–9 eggs/10 ml of urine, n = 49; odds ratio 1.4; 95% confidence interval [CI], 0.33–7.2; *P* = 0.75, by Fisher's exact test). Contemporaneous *P. falciparum* asexual parasite densities by microscopy were similar in both heavy and light *S. haematobium* infections (mean 140 parasites/µl blood; 95% CI, 1.6–280; mean 290 parasites/µl blood; 95% CI, −24–600; *P* = 0.37, by Welch's t test). Consistent with their recent anti-helminth treatment via MDA, only 31 (5.1%) of individuals had other helminthic infections at enrollment by stool microscopy with a single *S. mansoni* infection and 30 infections with the non-pathogenic intestinal helminth *Hymenolepis nana*. For real-time PCR diagnosis of additional helminth infections, only subsets of available samples were analyzed given the overall negative findings (Table 1). Additional baseline characteristics are shown in Table 1.

### Baseline Characteristics of *S. haematobium* Infected and Uninfected Individuals

Sex, HbAS, presence of mild anemia at enrollment, and presence of other helminthic infections were similarly distributed between *S. haematobium* infected and uninfected individuals (Table 1). The proportion of children with baseline *S. haematobium* infections increased with age (χ^2^ = 44.6, *P*<0.001 by Cochran-Armitage test for trend; Table 1). Individuals infected with *S. haematobium* were more likely to reside furthest away from both the health clinic and the main river in Kalifabougou (top tertile of distance from home to clinic or river) and were twice as likely to be infected with *P. falciparum* at enrollment by Fisher's exact test (unadjusted odds ratio = 2.0, *P* = 0.02; Table 1).

### Attrition Analysis

Of the 616 individuals who provided initial samples for this study, 560 (91%) completed follow up from May 2011 to January 2012. Among the 56 individuals who did not complete the study, 6 individuals (11%) had a clinical malaria episode with one death due to cerebral malaria. Those who remained free of malaria were censored at their last visit. The most common reasons for withdrawing were extended travel outside the study area (50%) and refusal of further blood draws (43%). Three women withdrew due to pregnancy. The attrition rate was highest in adults (3 months–2 years: 9%, 3–6 years: 8%, 7–8 years: 6%, 9–10 years: 9%, 11–17 years: 10%, 18–25 years: 40%; *P*<0.001). There was an increase in the attrition rate among individuals who were *S. haematobium*-infected at the time of enrollment (uninfected: 7%, *P. falciparum* mono-infection: 9%, *S. haematobium* mono-infection: 22%, co-infected: 15%; *P* = 0.056). Gender, spatial measures, sickle cell trait, anemia, and roof type were similarly distributed between those who did and did not complete the study.

### Spatial Analysis of Infections at Enrollment

**Figure 2:**
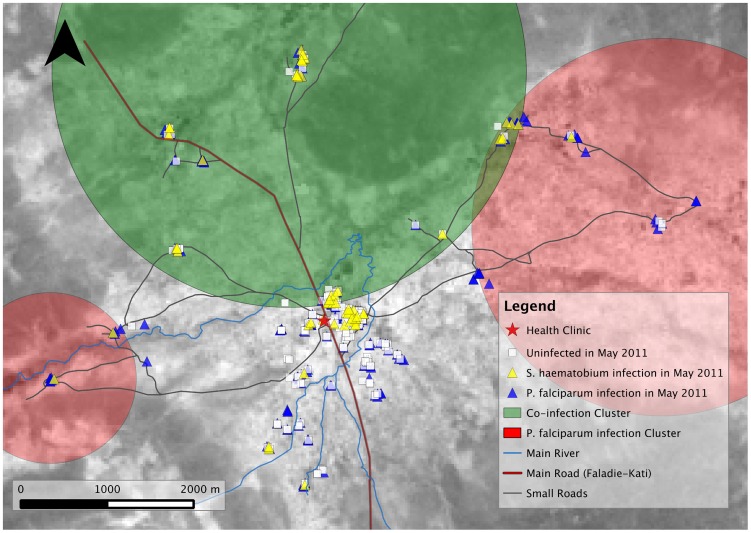
Spatial distribution of *S. haematobium* and *P. falciparum* infections in Kalifabougou, Mali at enrollment (May 2011). Shapes indicate infected and uninfected cases as noted. Large colored circles show significant, unadjusted clusters: green circle = cluster of co-infected cases in May 2011 (27 cases, n = 158, relative risk [RR] = 6.51, *P*<0.0001, Bernoulli model); red circles = clusters of *P. falciparum* infections in May 2011 (cluster 1: 35 cases, n = 41, RR = 1.90, *P*<0.001; cluster 2: 12 cases, n = 12, RR = 2.15, *P* = 0.04, Bernoulli model). Map data: Landsat image obtained from glovis.usgs.gov (latitude: 12.952, longitude: −8.173, imagery date: March 2011).

Geographical clustering may explain the disproportionate increase in *S. haematobium* infections and co-infections in areas furthest way from the clinic and river. We used SatScan as a tool for identifying geographical clusters that can be used as a proxy for unmeasured confounders related to *S. haematobium* infection and polyparasitism in regression models. The spatial distribution of PCR-positive *P. falciparum* infections, *S. haematobium* infections, co-infections, and uninfected controls at enrollment is shown in Figure 2. In May 2011, there was significant clustering of *S. haematobium* infected and co-infected individuals in an area centered ∼3 km north of the health clinic (28 cases, n = 94, relative risk [RR] = 4.57, *P*<0.0001; 27 cases, n = 158, RR = 6.51, *P*<0.0001, respectively). Both clusters overlapped substantially; therefore, only the co-infection cluster is shown in Figure 2. PCR-positive *P. falciparum* infections clustered significantly in two areas centered ∼4.6 km east-northeast (35 cases, n = 41, RR = 1.90, *P*<0.001) and ∼3.3 km west-southwest (12 cases, n = 12, RR = 2.15, *P* = 0.04) of the clinic (Figure 2).

### Baseline *Schistosoma haematobium* Infection and the Risk of *Plasmodium falciparum* Infection

**Figure 3:**
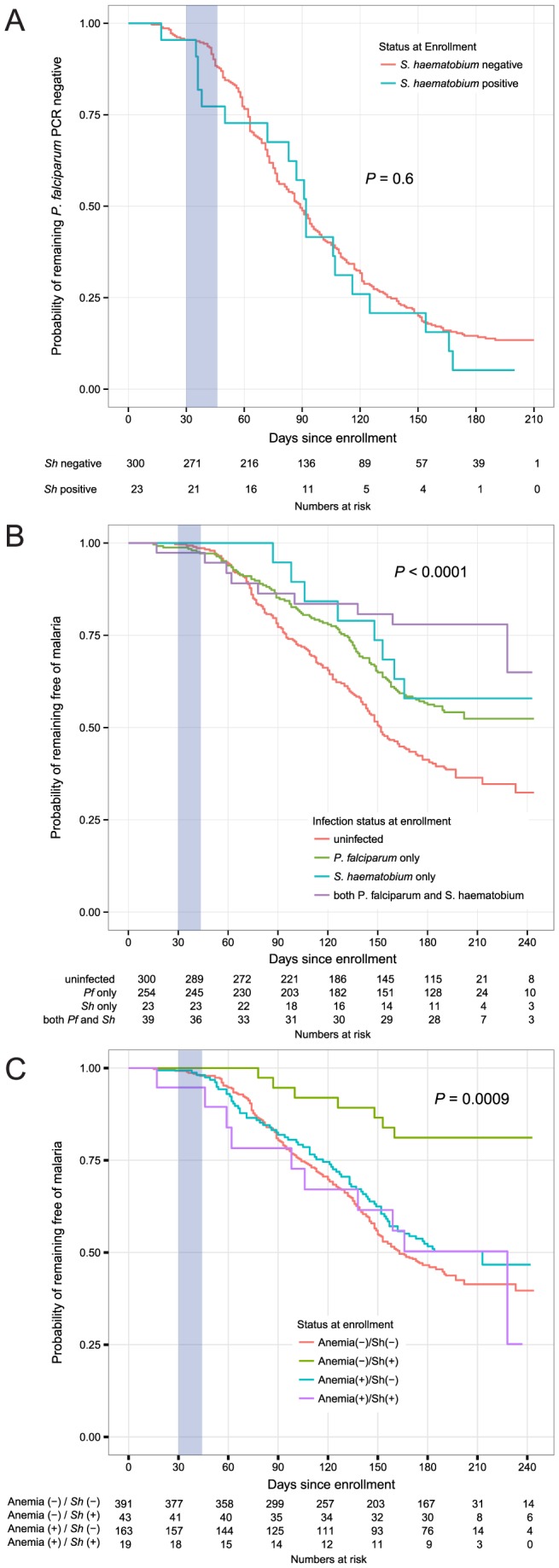
Kaplan-Meier plots of risk of *P. falciparum* infection or febrile malaria. A) Time to first PCR-confirmed *P. falciparum* blood-stage infection by *S. haematobium* (Sh) infection status at enrollment. Data shown is only for individuals who were PCR-negative for *P. falciparum* at enrollment. B) Time to first febrile malaria episode (defined as fever of ≥37.5°C and asexual parasite density ≥2500 parasites/µl on blood smear) by *P. falciparum* (Pf) and *S. haematobium* (Sh) infection status at enrollment. C) Time to first febrile malaria episode by *S. haematobium* (Sh) infection status and anemia status at enrollment. (−) negative status; (+) positive status. *P* values for log-rank analyses (all groups) are shown. Blue shading indicates time period during which praziquantel was given to all individuals who were determined to be infected with *S. haematobium* at enrollment.

Estimating the risk of *P. falciparum* blood-stage infection prospectively can be used as a surrogate of *P. falciparum* exposure [30]. Thus, we assessed the time to the first PCR-positive *P. falciparum* infection in individuals who began the study without *P. falciparum* infection and found no difference in the median time to *P. falciparum* PCR positivity between the *S. haematobium* uninfected and infected groups (89 days [95% confidence interval, CI, 81–96 days]; 92 days [95% CI, 83–125 days], respectively, *P* = 0.6, Figure 3A).

### Baseline *Schistosoma haematobium* and *Plasmodium falciparum* Infections and the Risk of Febrile Malaria

Given that asymptomatic *P. falciparum* carriage has been shown to affect the risk of febrile malaria [14], [15] and associates with *S. haematobium* infection [8], [9], [11], we estimated the risk of febrile malaria in individuals with 1) baseline *S. haematobium* mono-infection, 2) baseline *P. falciparum* mono-infection, 3) co-infection with both *P. falciparum* and *S. haematobium*, and 4) neither infection (uninfected). In the unadjusted analysis (Figure 3B), pairwise log-rank test between the uninfected group (median time to first malaria episode, 152 days [95% CI, 143–169 days]) and the 3 infected groups revealed significant delays in time-to-first malaria episode with *P. falciparum* mono-infection (median time not reached, *P*<0.001) and co-infection (median time not reached, *P*<0.001) but not with *S. haematobium* mono-infection (median time not reached, *P* = 0.054).

After adjustment for age, distance from home to river, HbAS, anemia, residence in the *S. haematobium* high-transmission cluster, and roof type in the Cox proportional hazards model, the protective effect of baseline *P. falciparum* mono-infection on febrile malaria persisted (hazards ratio [HR] = 0.71, 95% CI 0.55–0.92, *P* = 0.01; reference group: uninfected, Table 2). Baseline co-infection with *P. falciparum* and *S. haematobium* associated with enhanced protection from febrile malaria (HR = 0.44, 95% CI 0.22–0.90, *P* = 0.02; reference group: uninfected, Table 2), but the difference was not statistically significant relative to *P. falciparum* mono-infection (HR = 0.62, 95% CI 0.31–1.3, *P* = 0.19; reference group: *P. falciparum* mono-infection). Subset analysis of only individuals who were confirmed as negative for other helminth infections by stool PCR (Table 1, n = 142) revealed a similar association between co-infection and reduced malaria risk (HR = 0.20, 95% CI 0.06–0.71, *P* = 0.01; reference group: uninfected). Increased distance from the river was an independent predictor of malaria protection, while age, HbAS, and residence within the *S. haematobium* high-transmission cluster were associated with a non-significant trend towards reduced malaria risk at the parasite density threshold of ≥2500 asexual parasites/µl (Table 2). Metal roof houses have been previously shown to associate with reduced malaria risk, especially when they represent well-constructed housing [31], [32] as they do in Kalifabougou. However, we did not see any association between presence of a metal roof and malaria protection. Hazard ratio estimates of malaria risk using secondary definitions of malaria episodes (i.e. parasite density thresholds of any parasitemia, ≥500, and ≥5000 asexual parasites/µl) are shown in Table 2.

**Table 2:** Effect of baseline *Schistosoma haematobium* mono-infection, *Plasmodium falciparum* mono-infection, and co-infection on first or only malaria episode^a^.

		Parasite Density Threshold for Defining a Malaria Episode (number of events)															
		any parasitemia (363)				≥500 asexual parasites/µl (323)				≥2500 asexual parasites/µl (302)				≥5000 asexual parasites/µl (280)			
Variable	Subvariable	HR	lower 95% CL	upper 95% CL	*P*	HR	lower 95% CL	upper 95% CL	*P*	HR	lower 95% CL	upper 95% CL	*P*	HR	lower 95% CL	upper 95% CL	*P*
Age (per year increase)		0.97	0.94	1.0	0.02	0.97	0.95	1.0	0.07	0.97	0.95	1.0	0.08	0.97	0.94	1.0	0.03
Distance from home to river (per 100 m increase)		0.99	0.97	1.0	0.22	0.98	0.95	1.0	0.06	0.97	0.95	1.0	0.04	0.98	0.95	1.0	0.11
HbAS		0.8	0.55	1.2	0.26	0.73	0.48	1.1	0.14	0.66	0.43	1.0	0.07	0.65	0.41	1.0	0.07
Infection status at enrollment	Uninfected	REFERENCE				REFERENCE				REFERENCE				REFERENCE			
	*P. falciparum* mono-infection	0.79	0.62	1.00	0.05	0.71	0.55	0.91	0.008	0.71	0.55	0.92	0.01	0.68	0.52	0.89	0.004
	*S. haematobium* mono-infection	0.6	0.3	1.2	0.14	0.57	0.28	1.2	0.13	0.62	0.3	1.3	0.20	0.56	0.26	1.2	0.14
	Co-infection	0.94	0.56	1.6	0.82	0.54	0.29	1.0	0.06	0.44	0.22	0.90	0.02	0.48	0.24	0.98	0.04
Mild anemia at baseline		0.95	0.75	1.2	0.66	0.82	0.64	1.1	0.14	0.83	0.63	1.1	0.16	0.79	0.6	1.0	0.09
Residence in *S. haematobium* transmission cluster		0.64	0.43	0.95	0.03	0.72	0.47	1.1	0.12	0.66	0.42	1.0	0.07	0.72	0.45	1.1	0.15
Metal roof		1.1	0.86	1.3	0.57	0.97	0.77	1.2	0.79	0.96	0.76	1.2	0.77	1.0	0.81	1.3	0.79

Abbreviations: CL, confidence limit; HR, hazard ratio; HbAS, sickle cell trait.

a Risk of first or only malaria episode was adjusted for age, distance from home to river, sickle cell trait, anemia status at baseline, residence in the cluster of high *S. haematobium* transmission, and roof type in the classic Cox proportional hazards model.

We found a significant interaction between anemia and *S. haematobium* infection, as the protective effect of *S. haematobium* was apparent only in individuals without anemia (Figure 3C). Inclusion of an interaction term between anemia and the two *S. haematobium* infected groups in the Cox model strengthened the association between baseline co-infection with protection from febrile malaria (HR = 0.14, 95% CI 0.034–0.57, *P* = 0.006; reference group: uninfected, Table 3), and notably, co-infection was significantly more protective than *P. falciparum* mono-infection (HR = 0.19, 95% CI 0.05–0.80, *P* = 0.02; reference group: *P. falciparum* mono-infection).

**Table 3:** Effect of baseline *Schistosoma haematobium* mono-infection, *Plasmodium falciparum* mono-infection, and co-infection on first or only malaria episode (with anemia interaction term)^a^.

		Parasite Density Threshold for Defining a Malaria Episode (number of events)															
		any parasitemia (363)				≥500 asexual parasites/µl (323)				≥2500 asexual parasites/µl (302)				≥5000 asexual parasites/µl (280)			
Variable	Subvariable	HR	lower 95% CL	upper 95% CL	*P*	HR	lower 95% CL	upper 95% CL	*P*	HR	lower 95% CL	upper 95% CL	*P*	HR	lower 95% CL	upper 95% CL	*P*
Age (per year increase)		0.97	0.94	0.99	0.01	0.97	0.95	1.0	0.07	0.97	0.95	1.0	0.08	0.97	0.94	1.0	0.03
Distance from home to river (per 100 m increase)		0.99	0.97	1.0	0.23	0.98	0.96	1.0	0.07	0.97	0.95	1.0	0.05	0.98	0.96	1.0	0.13
HbAS		0.8	0.55	1.2	0.25	0.73	0.49	1.1	0.14	0.67	0.43	1.0	0.07	0.66	0.41	1.0	0.07
Infection status at enrollment	Uninfected	REFERENCE				REFERENCE				REFERENCE				REFERENCE			
	*P. falciparum* mono-infection	0.79	0.62	1.0	0.05	0.71	0.55	0.91	0.007	0.71	0.55	0.92	0.009	0.67	0.51	0.88	0.004
	*S. haematobium* mono-infection	0.47	0.21	1.1	0.08	0.41	0.17	1.0	0.06	0.45	0.18	1.1	0.09	0.36	0.13	0.99	0.048
	Co-infection	0.39	0.17	0.92	0.03	0.25	0.091	0.69	0.008	0.14	0.034	0.57	0.006	0.14	0.035	0.59	0.007
Mild anemia at baseline		0.83	0.65	1.1	0.16	0.75	0.57	0.98	0.03	0.75	0.57	0.99	0.04	0.7	0.52	0.93	0.02
Residence in *S. haematobium* transmission cluster		0.68	0.46	1.0	0.05	0.74	0.49	1.1	0.15	0.68	0.43	1.1	0.09	0.74	0.47	1.2	0.2
Metal roof		1.1	0.88	1.4	0.41	0.98	0.78	1.2	0.88	0.98	0.77	1.2	0.86	1.1	0.82	1.4	0.69
Anemia*co-infection		6.1	2.2	17	0.0004	5.0	1.5	17	0.01	8.4	1.7	41	0.009	9.3	1.9	46	0.006
Anemia*S. haematobium mono-infection		2.3	0.55	9.2	0.26	3.3	0.77	14	0.11	3.3	0.76	14	0.11	4.6	1.0	21	0.05

Abbreviations: CL, confidence limit; HR, hazard ratio; HbAS, sickle cell trait.

a Risk of first or only malaria episode was adjusted for age, distance from home to river, sickle cell trait, anemia status at baseline, residence in the cluster of high *S. haematobium* transmission, and roof type in the classic Cox proportional hazards model with inclusion of interaction terms between anemia status and the two covariates with *S. haematobium* infection (anemia*co-infection and anemia*S. haematobium mono-infection).

### *Plasmodium falciparum* Parasite Density at the First Febrile Malaria Episode

Multiple linear regression analysis of parasite density at the first febrile malaria episode revealed that increasing age and asymptomatic *P. falciparum* carriage were strong negative predictors of parasite density, whereas light *S. haematobium* mono-infection (1–9 eggs/10 ml urine) had no effect on parasite density at the first malaria episode (Table 4). Interestingly, individuals with heavy *S. haematobium* mono-infection (≥10 eggs/10 ml urine) only suffered from febrile malaria episodes with parasite densities of <500 parasites/µl, and among those episodes, heavy *S. haematobium* negatively predicted parasite density (Table 4). Baseline co-infection with both *P. falciparum* and *S. haematobium* was a significant, independent predictor of lower *P. falciparum* parasite density at all definitions for malaria except for the malaria case definition (≥2500 asexual parasites/µl) (Table 4).

**Table 4:** Multiple linear regression model of parasite density at the first febrile malaria episode by different parasite density thresholds^a^.

		Parasite Density Threshold for Defining a Malaria Episode (number of events)															
		any parasitemia (363)				≥500 asexual parasites/µl (323)				≥2500 asexual parasites/µl (302)				≥5000 asexual parasites/µl (280)			
Explanatory variable	Subvariable	Coefficient	lower 95% CL	upper 95% CL	*P*	Coefficient	lower 95% CL	upper 95% CL	*P*	Coefficient	lower 95% CL	upper 95% CL	*P*	Coefficient	lower 95% CL	upper 95% CL	*P*
log(age in years)		−0.0096	−0.32	0.31	0.95	−0.24	−0.45	−0.02	0.03	−0.25	−0.42	−0.07	0.007	−0.25	−0.41	−0.09	0.002
log (meters from home to clinic)		−0.13	−0.43	0.17	0.40	−0.03	−0.25	0.18	0.77	0.06	−0.12	0.24	0.51	−0.01	−0.17	0.15	0.88
HbAS		−0.48	−1.32	0.37	0.27	−0.14	−0.72	0.44	0.64	0.09	−0.39	0.58	0.71	0.10	−0.35	0.54	0.67
Infection status at enrollment	Uninfected	REFERENCE				REFERENCE				REFERENCE				REFERENCE			
	*P. falciparum* mono-infection	−0.63	−1.14	−0.11	0.02	−0.34	−0.68	0.00	0.05	−0.39	−0.67	−0.11	0.006	−0.18	−0.43	0.07	0.15
	Light *S. haematobium* mono-infection^b^	0.85	−0.76	2.46	0.30	−0.09	−1.10	0.93	0.87	−0.26	−1.06	0.54	0.52	−0.16	−0.91	0.58	0.67
	Heavy *S. haematobium* mono-infection^c^	−4.47	−8.90	−0.04	0.048	NA	NA	NA	NA	NA	NA	NA	NA	NA	NA	NA	NA
	Co-infection	−1.77	−2.93	−0.60	0.003	−1.23	−2.11	−0.36	0.01	−0.68	−1.47	0.12	0.10	−0.79	−1.48	−0.10	0.03
Mild anemia at baseline		−0.21	−0.75	0.33	0.45	0.17	−0.19	0.54	0.35	0.08	−0.22	0.38	0.59	0.21	−0.06	0.48	0.12
Residence in *S. haematobium* transmission cluster		−0.04	−1.01	0.92	0.93	−0.18	−0.83	0.47	0.587	−0.06	−0.62	0.50	0.833	−0.17	−0.66	0.32	0.49

Abbreviations: CL, confidence limit; HbAS, sickle cell trait; NA = not assessed due to lack of individuals with heavy *S. haematobium* mono-infection in analysis.

a Effect of infection status at enrollment on parasite density in log(parasites/µl) using a general linear model with adjustments for age, distance from home to clinic, sickle cell trait, baseline anemia status, and residence in the cluster of high *S. haematobium* transmission.

b 1–9 eggs/10 mL urine.

c ≥10 eggs/10 ml urine.

## Discussion

Investigating the relationship between different parasitic infections in co-endemic communities at the population level is challenging due to the possibility of confounding by unknown variables that co-associate with both diseases. The interaction between *P. falciparum* and *S. haematobium* is one relationship where evaluating confounders may help explain inconsistent findings in the literature. In this prospective cohort study of malaria risk, we accounted for several possible confounders and observed that *S. haematobium* infection enhances protection from febrile malaria in individuals with asymptomatic *P. falciparum* carriage.

In this study, asymptomatic *P. falciparum* carriers were more likely to be co-infected with *S. haematobium* at enrollment, which corroborates previous cross-sectional studies [8], [9], [11]. Prospective, univariate analysis demonstrated that both baseline *P. falciparum* and *S. haematobium* mono-infections predict protection from malaria, with stronger significance for *P. falciparum* than *S. haematobium* (Figure 3B), possibly due to a difference in statistical power (i.e. there was a low number of *S. haematobium* mono-infections). Taken separately, both of these findings are consistent with previous studies done in areas of seasonal malaria transmission [10], [14], [15].

Notably, co-infection with both parasites conferred greater protection from subsequent febrile malaria (Figure 3B), a finding that, to our knowledge, has not been reported elsewhere. To further investigate this finding, we performed an adjusted analysis of malaria risk in which we included covariates that could potentially affect malaria outcomes based on prior studies (age, HbAS, anemia) or that were differentially distributed between individuals with and without *S. haematobium* at baseline (age, distance from home to river, residence within a high-transmission cluster). In the adjusted model, asymptomatic *P. falciparum* carriage and co-infection, but not *S. haematobium* mono-infection, independently predicted protection from febrile malaria (Tables 2 and 3).

Given that we stopped screening individuals for gastrointestinal helminths by stool PCR at only 23% of the cohort due to completely negative findings (Table 1), it is possible that baseline co-infection with these and other helminths among unscreened individuals confounded our findings. However, the malaria-protective effect of *S. haematobium* and *P. falciparum* co-infection persisted even when the same regression analysis was restricted to stool-negative individuals (n = 142), suggesting that co-infections with gastrointestinal helminths are unlikely to confound our interpretation of the data. Our data demonstrates that heavy *S. haematobium* infections at baseline predict lower *P. falciparum* parasite densities at the first malaria episode, suggesting a potential negative interaction between the two parasites. However, we did not observe differences in malaria risk by intensity of *S. haematobium* infection, perhaps due to the low prevalence of heavy *S. haematobium* infections in our study (2.1%), a finding consistent with recent praziquantel MDA.

The above findings may help reconcile the disparity between two previous prospective cohort studies of *S. haematobium* infection and malaria risk. A study conducted in Mali reported an association between baseline *S. haematobium* infection and protection from malaria attacks but did not differentiate between mono-infection and co-infection [10]. Conversely, *S. haematobium* mono-infection did not influence malaria risk in a malaria vaccine efficacy study conducted in Kenya in which all children were cleared of *P. falciparum* with anti-malarials immediately prior to surveillance [13]. It is important to note, however, in both our study and the Kenyan study, the frequencies of *S. haematobium* mono-infections were small (3.7% and 8%, respectively), suggesting limited power to detect a difference in malaria risk for this group. Indeed, with only 23 cases of *S. haematobium* mono-infections, we had a 32% probability of detecting a hazard ratio of 0.62 or smaller at a 2-sided significance level of 0.05. While we might have detected more light infections had we examined more than one urine specimen per individual or used a more sensitive molecular diagnostic assay [33], it is evident that additional studies with larger sample sizes, perhaps in an area of higher *S. haematobium* prevalence, are needed to better address whether *S. haematobium* mono-infection confers protection from febrile malaria *per se* and also if infection intensity might affect malaria risk.

A plausible mechanism of how co-infection enhances the protection conferred by asymptomatic *P. falciparum* carriage against febrile malaria is suggested by prior studies which demonstrated increased production of the anti-inflammatory cytokine IL-10 in co-infected individuals relative to individuals infected with only *P. falciparum* by either analysis of circulating plasma cytokines [34] or after in vitro stimulation of peripheral blood mononuclear cells with *P. falciparum* schizont extract [35]. In addition, we have observed that *P. falciparum*-inducible IL-10 responses are upregulated in asymptomatic children with chronic *P. falciparum* infections [36]. Thus, *S. haematobium* infection could further augment anti-inflammatory responses induced by asymptomatic *P. falciparum* infection, thereby blunting the risk of fever upon subsequent *P. falciparum* infections.

Curiously, baseline co-infection predicted protection from febrile malaria despite treatment with the anti-schistosomal agent praziquantel (Figure 3B). This suggests that the putative immunomodulatory effects of *S. haematobium* persist for an unknown period of time following clearance of *S. haematobium*. Although speculative, it is plausible that co-infection induces epigenetic changes that maintain an anti-inflammatory environment—a mechanism described in a mouse model of *S. mansoni* infection at the level of alternatively activated macrophages [37]. However, several alternative possibilities could also explain the protective association of co-infection with malaria risk despite praziquantel therapy. Individuals who were infected at baseline may simply be at the highest risk for re-infection after treatment. An important limitation of our study is that we were not able repeat surveillance for urogenital schistosomiasis to determine the re-infection frequencies in our cohort. Since we also did not confirm parasite clearance after administration of praziquantel, modulation of host responses by persistent *S. haematobium* infection due to ineffective killing of juvenile worms (a known limitation of praziquantel) or complete drug failure remains a possibility. The latter is less likely given that, to our knowledge, there have been no reports of praziquantel failure in Mali since the initiation of MDA. An immunomodulatory effect of praziquantel *per se* is another possibility, but this is not supported by our data given the more modest protection seen in individuals who received praziquantel for *S. haematobium* mono-infection during the same time period as co-infected individuals (Table 2). Lastly, unknown genetic, behavioral, or environmental factors that co-associate specifically with co-infected individuals and reduced malaria risk may be confounding the findings of this study.

We were intrigued by the observation that those individuals living the furthest from the clinic and river were more likely to be infected with *S. haematobium* at baseline and hypothesized that geographical clustering may explain this finding. Indeed, spatial cluster analysis of infections at the time of enrollment clearly demonstrated a significant cluster of *S. haematobium*-infected and co-infected individuals in an area north of the clinic, where a striking 28 of the 62 (45%) *S. haematobium* cases reside. Here, we used SatScan solely for identifying geographical clusters of disease that can then be used as a proxy for unmeasured confounders in regression models, noting that the spatial statistic employed by SatScan operates ideally when disease information is known for all households rather than a sampling of households. Nevertheless, these findings support a previous study in Kenya which found that intense infections of *P. falciparum* and *S. haematobium* clustered together within a subset of individuals even after the authors controlled for behavioral factors related to exposure to both parasites, implicating host susceptibility factors as the reason for this phenomenon [9]. By including residence in the *S. haematobium* transmission cluster as a covariate in our models, we were thus able to adjust for geospatial factors related to *S. haematobium* infection that we could not directly measure, such as host susceptibility factors, unmapped water sources serving as co-infective reservoirs, and micro-heterogeneity of malaria exposure, the last which has been shown to occur at sites similar to Kalifabougou [38], [39]. However, comparable rates of *P. falciparum* blood-stage infection between individuals with and without baseline *S. haematobium* infection (Figure 3A) would suggest that heterogeneity of malaria exposure is less likely to be a confounder of *S. haematobium* infection and malaria risk.

Reduced malaria risk was seen almost exclusively in *S. haematobium*-infected individuals without anemia (Figure 3C), and a significant interaction between the two variables was observed in one model of malaria risk (Table 3). These findings may be related to the intensity of *S. haematobium* infection, as more severe egg burden has been associated with increased hematuria and anemia [40] as well as increased *P. falciparum* density [9]. We did not observe an association between infection intensity and anemia in our study, although this may simply be a reflection of the low prevalence of heavy *S. haematobium* infections in our study. More likely, anemia may simply be a marker of malnutrition [41], which has been independently associated with increased malaria risk [42] and thus may minimize any malaria-protective effect conferred by *S. haematobium* infection.

Potential sources of bias are worth noting. Although we randomly sampled in an age-stratified manner from the entire village-wide census, we enrolled only healthy, afebrile individuals. Thus, we could have excluded individuals with symptomatic infections (including *P. falciparum* and/or *S. haematobium* infection), and it is possible that these individuals would be the most susceptible to subsequent malaria episodes. However, since only 4% of screened individuals were excluded due to fever, the potential for bias would be minor. An addition source of bias may be from the higher attrition rates among the *S. haematobium*-infected individuals (18%), as these individuals may have developed malaria had they remained in the study. This potential bias is mitigated by the fact that 55% of the *S. haematobium*-infected individuals who withdrew from the study were adults and thus would be less likely to have a malaria episode, and three individuals completed more than 6 months of follow up prior to withdrawal.

In summary, we conducted a prospective cohort study to investigate the relationship between *S. haematobium* and *P. falciparum* infection and the risk of febrile malaria that accounted for several biological and contextual variables. We observed that *S. haematobium* co-infection is associated with enhanced protection from febrile malaria in long-term asymptomatic carriers of *P. falciparum*. Future studies are needed to investigate whether co-infected individuals share other genetic, behavioral, or environmental factors not included here that may explain this association. In addition, further studies are needed to understand the immunological state induced by co-infection and its impact on clinical outcomes of *P. falciparum* infection.

## Supporting Information

Checklist S1 **STROBE checklist.**
(DOC)
